# Platypnea and orthodeoxia associated with *Pneumocystis jiroveci *and *Cytomegalovirus *pneumonia: a case report

**DOI:** 10.1186/1752-1947-3-9319

**Published:** 2009-12-05

**Authors:** Konstantinos Katsoulis, Ilias Minasidis, Andreas Vainas, Christoforos Bikas, Theodoros Kontakiotis, Pantelis Vakianis

**Affiliations:** 1 Pulmonary Department, General Army Hospital, Thessaloniki, Greece; 2 Nephrology Department, General Army Hospital, Thessaloniki, Greece

## Abstract

**Introduction:**

Platypnea-orthodeoxia is an uncommon syndrome characterized by dyspnea and deoxygenation accompanying a change to a sitting or standing posture from a recumbent position. It is usually related to interatrial communications, although several other disorders associated with platypnea-orthodeoxia syndrome have been reported. However, the precise mechanisms are unknown.

**Case presentation:**

We present the case of a 75-year-old Caucasian woman with chronic renal failure due to vasculitis who was admitted with fever and respiratory failure. She was found to have both *Pneumocystis jiroveci *and *Cytomegalovirus *pneumonia. She was HIV negative. Severe platypnea and orthodeoxia were major features of her illness with no history of respiratory, liver or cardiac disease. Further investigation with contrast echocardiography revealed no intracardiac or intrapulmonary shunts. Although one case involving *Pneumocystis jiroveci *pneumonia and platypnea has been previously reported, to the best of our knowledge, this is the first time that two opportunistic pathogens have been accompanied by platypnea and orthodeoxia. As both lung bases were predominantly affected and no obvious explanation was found, platypnea and orthodeoxia were attributed to significant areas of low or zero ventilation/perfusion (V/Q) ratio.

**Conclusion:**

Platypnea-orthodeoxia is a rare and usually underestimated syndrome. Intracardiac shunts and anatomic pulmonary vascular shunts are the most common etiologic associations. However, if a detailed examination reveals no obvious intracardiac or intrapulmonary shunting combined with extensive pulmonary lesions, then severe V/Q mismatching should be considered as the probable explanation.

## Introduction

Platypnea-orthodeoxia is a relatively uncommon but striking clinical syndrome characterized by dyspnea and deoxygenation accompanying a change to a sitting or standing posture from a recumbent position. It was first reported in 1949 when Burchell *et al*. [1] described a patient with an atrial septal defect manifesting platypnea-orthodeoxia and subsequently described the reversal of both following closure of a patent foramen ovale. 'Platypnea' and 'orthodeoxia' were not used to describe the manifestations of this syndrome until they became commonly accepted terms in 1969 and 1976, respectively [2,3]. Since then, a few cases have been reported with interatrial communications being the most common etiologic associations [4-6]. The precise mechanisms for both platypnea and orthodeoxia are unknown. In several isolated case reports, speculation over mechanisms is often geared to whatever special features were found in the patient been reported. We present a case of a patient with severe platypnea and orthodeoxia infected with two opportunistic pathogens and with no evidence of intracardiac or intrapulmonary shunt.

### Case presentation

A 75-year-old Caucasian woman was admitted to our hospital with febrile illness accompanied by dyspnea without other specific symptoms, such as cough or sputum. She was normotensive, and her heart sounds were normal with bibasal lung crepitations. An electrocardiogram demonstrated sinus rhythm with a normal axis and oxygen saturation in room air was 75%. Platypnea and orthodeoxia were major features of her illness. When supine on 35% oxygen by face mask, arterial blood gas measurements yielded persistent hypoxemia (pO_2_: 70 mmHg, pCO_2_: 30 mmHg) with counterbalanced metabolic acidosis (HCO_3_: 18 mmol/l, pH 7.44). However, in the upright position, she developed severe hypoxemia (pO_2_: 40 mmHg). Biochemical tests showed renal failure (urea: 100 mg/dl, creatinine: 2 mg/dl, hematocrit (Hct): 30%), increased lactate dehydrogenase (LDH) levels (600 U/L) and increased markers of inflammation (erythrocyte sedimentation rate (ESR): 100 mm, CRP: >100 mg/dl), while a chest X-ray showed a few bilateral diffuse interstitial infiltrates, predominantly in the lower lobes.

Five months before admission, oliguric acute renal failure was detected and kidney biopsy revealed rapidly progressive glomerulonephritis with 100% crescents compatible with Wegener's disease or nodular polyarteritis. She was initially treated with sessions of renal dialysis and plasmapheresis combined with pulses of methylprednisolone. Afterwards, the treatment switched to oral methylprednisolone at 48 mg/day combined with oral cyclophosphamide at 100 mg/day for 2 months with progressive lessening of the doses. Cyclophosphamide was finally withdrawn due to severe side effects (leucopenia). During the last trimester, she was in good condition under treatment with 16 mg/day of methylprednisolone.

**Figure 1:**
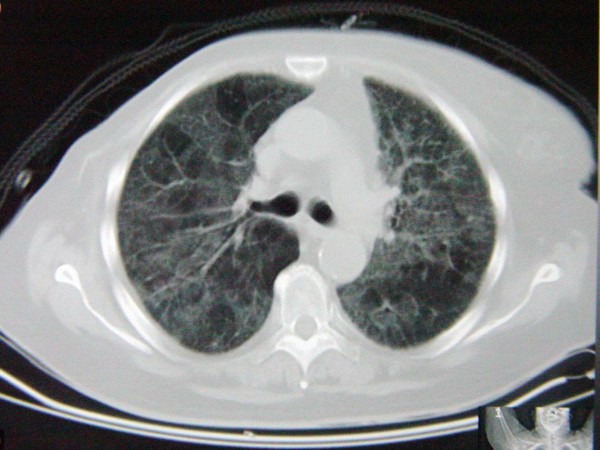
**Computed tomography demonstrating patchy areas of ground-glass opacity**.

She was initially treated with empirical antibiotic treatment and underwent computed tomographic (CT) scanning which showed patchy areas of ground-glass opacity (Figure 1). With the suspicion of *Pneumocystis jiroveci *pneumonia and despite a negative test for HIV, fiberoptic bronchoscopy and bronchoalveolar lavage (BAL) were performed. Immunostaining of the specimens was positive for *P. jiroveci *and several cysts were microscopically visualized. Thus, the treatment was changed to high-dose intravenous co-trimoxazole and prednisolone.

**Figure 2:**
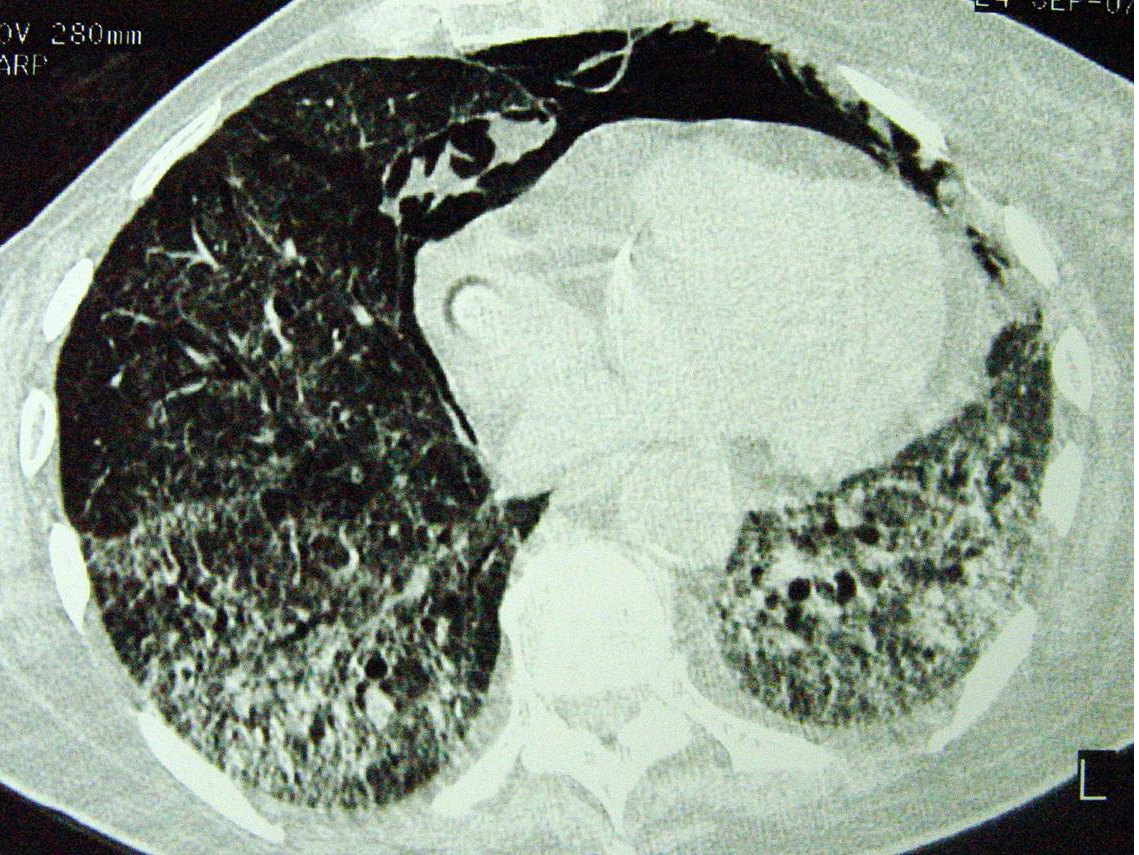
**Computed tomography demonstrating organized consolidations with air bronchogram at the lung bases and air in the mediastinum**.

Although she became afebrile, the clinical presentation deteriorated with excessive platypnea coupled with orthodeoxia. Sitting up was associated with a fall in her oxygen saturation of up to 67% under oxygen administration. As there was no evidence of liver disease or hepatopulmonary syndrome, a transthoracic echocardiogram was performed. Intravenously administered normal saline was not detected in the left atrium after two or six cardiac circles excluding the presence of intracardiac or intrapulmonary shunts. Due to the respiratory distress, a pulmonary angiogram was not performed. A new CT scan revealed further deterioration consisting of organized consolidations with air bronchogram at the lung bases and air in the mediastinum (Figure 2). On day 15, she was intubated and admitted to the intensive care unit. At the same time, BAL examination was positive for cytomegalovirus (CMV) through polymerase chain reaction (PCR) (23,000 copies/ml). Intravenous ganciclovir (5 mg/kg, twice a day) was added to the treatment. Despite the appropriate treatment, she died on day 25.

## Discussion

Platypnea (increased dyspnea in the erect position relieved by assuming a recumbent position) and orthodeoxia (accentuated hypoxemia in the erect position, improved by assuming a recumbent position) were the patient's more striking symptoms. Only one case of this syndrome associated with *Pneumocystis carinii *pneumonia (the old term now replaced by *jiroveci*) has been previously reported [7]. However, to the best of our knowledge, the association of this syndrome with pulmonary infection by two opportunistic pathogens, including *P. jiroveci*, has not been reported.

The etiologic background of this syndrome includes intracardiac shunts, anatomic pulmonary vascular and pulmonary parenchymal shunts, with interatrial communications being the most common etiologic association, but the precise mechanisms are not known [5,8,9]. Other diseases associated with platypnea-orthodeoxia include chronic obstructive pulmonary disease (COPD) [10], constrictive pericarditis [11], pneumonectomy [12], paradoxical embolism [13] and even acute organophosphorus poisoning [14].

In our patient, *P. jiroveci *and CMV were detected in BAL specimens using reliable methods (immunostaining/microscopic visualization and PCR, respectively) in an immunosuppressed patient. The radiological findings were not diagnostic as both of the above pathogens present with similar findings. It has been noted that coinfection with CMV and other pathogens will be detected in more than half of *Pneumocystis*-infected patients [15] and thus the major pathogen is not easily defined. As we found no other common pathogens and our patient continued to deteriorate despite the appropriate treatment for *P. jiroveci *pneumonia, we believe that CMV was responsible for the fatal deterioration.

Our patient had no history or evidence of COPD or chronic liver disease (for example, cirrhosis). As platypnea and orthodeoxia were major features of her illness, further investigation focused on the detection of probable intracardiac or intrapulmonary shunts using contrast echocardiography, a widely accepted and non-invasive method [16]. No evidence of such disorders was found, so these symptoms probably arose as a result of significant areas of low or zero V/Q ratio. Indeed, a CT scan indicated that the lung bases were predominantly affected. Gravity might increase intrapulmonary blood flow shunting through poorly ventilated lung bases exacerbating dyspnea and deoxygenation in the upright position. The same explanation has been proposed by other authors for similar cases [5,7].

Whether specific pathogens such as *P. jiroveci *and/or CMV or severe V/Q mismatching of any etiology are responsible for the emergence of this syndrome remains to be clarified.

## Conclusion

Platypnea-orthodeoxia is a rare and usually underestimated syndrome. Intracardiac shunts and particularly, interatrial communications with or without overt lung disease as well as anatomic pulmonary vascular shunts are the most common etiologic associations. However, if detailed examination reveals no obvious intracardiac or intrapulmonary shunting combined with extensive pulmonary lesions, such as severe pneumonia even due to opportunistic pathogens, then severe V/Q mismatching should be considered as the probable explanation.

## Abbreviations

BAL: bronchoalveolar lavage; COPD: chronic obstructive pulmonary disease; CMV: cytomegalovirus; CRP: C-reactive protein; ESR: erythrocyte sedimentation rate; Hct: hematocrit; LDH: lactate dehydrogenase; PCR: polymerase chain reaction; V/Q: ventilation/perfusion.

## Consent

Written informed consent was obtained from the patient for publication of this case report and accompanying images. A copy of the written consent is available for review by the Editor-in-Chief of this journal

## Competing interests

The authors declare that they have no competing interests.

## Authors' contributions

KK was the main author and carried out the pulmonary investigation of the case. IM carried out the nephrological investigation of the case. AV carried out vasculitis diagnosis and management of the case. CB carried out the respiratory failure diagnosis and management. TK is the corresponding author and was responsible for manuscript preparation and the pulmonary investigation of the case. PV carried out the renal failure diagnosis and management of the case.

## References

[B1] Burchell HB Hemholz HF Jr Wood EH Reflect orthostatic dyspnea associated with pulmonary hypotension Am J Physiol 1949 159 563 564

[B2] Altman M Robin ED Platypnea (diffuse zone I phenomenon?) N Engl J Med 1969 281 1347 1348 10.1056/NEJM196912112812408 5355439

[B3] Robin ED Lamon D Horn BR Theodore J Platypnea related to orthodeoxia caused by true vascular lung shunts N Engl J Med 1976 294 941 943 10.1056/NEJM197604222941711 1256486

[B4] Seward JB Hayes DL Smith HC Williams DE Piehler JM Tajik AJ Platypnea-orthodeoxia: clinical profile, diagnostic workup, management and report of seven cases Mayo Clin Proc 1984 59 4 221 231 10.1016/s0025-6196(12)61253-1 6708599

[B5] Robin ED McCauley F An analysis of platypnea-orthodeoxia syndrome including a "new" therapeutic approach Chest 1997 112 1449 1451 10.1378/chest.112.6.1449 9404736

[B6] Gobart F Rey C Platypnea-orthodeoxia syndrome: a probably underestimated syndrome? Chest 2001 119 1624 1625 10.1378/chest.119.5.1624-a 11348988

[B7] Newton PN Wakefield AE Goldin R Govan J Pneumocystis carinii pneumonia with pleurisy, platypnoea and orthodeoxia Thorax 2003 58 185 186 10.1136/thorax.58.2.185 PMC1746571 12554907

[B8] Hagen PT Scholz DG Edwards WD Incidence and size of patent foramen ovale during the first ten decades of life: an autopsy study of 965 normal hearts Mayo Clin Proc 1984 59 17 20 10.1016/s0025-6196(12)60336-x 6694427

[B9] Cheng TO Mechanisms of platypnea-orthodeoxia: what causes water to flow uphill? Circulation 2002 105 6 47 11839642

[B10] Hussain SF Mekan SF Platypnea-orthodeoxia: report of two cases and review of the literature South Med J 2004 97 7 657 662 10.1097/00007611-200407000-00010 15301123

[B11] Hashimoto M Okawa Y Baba H Nishimura Y Aoki M Platypnea-orthodeoxia syndrome combined with constrictive pericarditis after coronary artery bypass surgery J Thorac Cardiovasc Surg 2006 132 5 1225 1226 10.1016/j.jtcvs.2006.07.021 17059949

[B12] Kotoulas C Patris K Tsintiris K Zoumboulides A Lazarides K Laoutides G Platypnea-orthodeoxia syndrome after pneumonectomy relieved by mediastinal repositioning Ann Thorac Surg 2007 83 4 1524 1526 10.1016/j.athoracsur.2006.11.004 17383374

[B13] Delalieux S De Greef K Hendriks J Lauwers P Suys B Van Schil P Orthodeoxia-platypnea syndrome presenting as paradoxical peripheral embolism Ann Thorac Surg 2008 85 5 1798 1800 10.1016/j.athoracsur.2007.08.011 18442594

[B14] Bouros D Agouridakis P Tsatsakis A Askitopoulou E Siafakas NM Orthodeoxia and platypnoea after acute organophosphorus poisoning reversed by CPAP: a newly described cause and review of the literature Respir Med 1995 89 9 625 628 10.1016/0954-6111(95)90232-5 7494917

[B15] Fishman JA Pneumocystis carinii Fishman's Pulmonary Diseases and Disorders 1998 3 McGraw-Hill 2313 2331

[B16] Thakur CT Nanda NC Malhotra S St Martin MB Jamil F Agrawald D Maheshwari S Abrams GA Patel B Combined interatrial and intrapulmonary shunting in orthodeoxia detected by transoesophageal echocardiography Echocardiography 2007 15 101 104 10.1111/j.1540-8175.1998.tb00584.x 11175017

